# Barriers to accessing eye care in Pakistan: a mixed methods study

**DOI:** 10.1017/S1463423625100261

**Published:** 2025-07-15

**Authors:** Manal Malik, Niall Strang, Aiman Hafeez, Mujtaba Shabbir, Farah Iftikhar, Sven Jonuscheit

**Affiliations:** 1 Department of Vision Sciences, Glasgow Caledonian University, Govan Mbeki Building, Glasgow G4 0BA, UK; 2 School of Health and Psychological Sciences, Department of Optometry and Visual Science, City University of London, London EC1V 0HB, UK; 3 Adam Smith Business School, University of Glasgow, Glasgow G12 8QQ, UK; 4 Department of Optometry, Trauma Centre, Tehsil Head Quarter Kalarkahar, Chakwal, Pakistan

**Keywords:** Eye care access, healthcare delivery, mixed methods, Pakistan, Sehat Sahulat programme, universal health coverage, visual impairment

## Abstract

**Aims:**

To support policymakers in enhancing access to eye care for the population aged 45 years and older in Pakistan, this study aims to identify and quantify the barriers that hinder effective eye care delivery to this group. Additionally, it seeks to explore patients’ experiences with the Sehat Sahulat (health insurance) programme in the context of eye care services.

**Background:**

Accessible eye care services can reduce avoidable blindness by delivering timely, high-quality interventions. In Pakistan, the lack of primary eye care burdens overcrowded hospitals and combined with economic challenges, limits access for underprivileged populations. To address this, a nationwide health insurance scheme – the Sehat Sahulat programme (SSP) was introduced to reduce out-of-pocket (OOP) expenses and improve healthcare access for economically disadvantaged groups.

**Methods:**

Using an exploratory sequential mixed methods design, an initial qualitative phase explored participant experiences and identified specific barriers. The qualitative study provided the basis for the development of a customized survey tool. The survey tool was then used in a second phase to obtain quantitative data to capture the magnitude of barriers and costs associated with accessing eye care in Pakistan.

**Findings:**

Numerous considerable barriers were identified including illiteracy, long travel times, female gender, old age, mobility issues, and costs, all of which limited access to eye care in Pakistan. Awareness surrounding use of the SSP was poor, with the programme seldom used towards eye care costs. This study highlights patient experiences with eye care in urban and rural Pakistan, including enablers and barriers to accessing eye care. Improvements should focus on educating the public on eye health, increasing availability of eye care services in rural areas, improving accessibility within eye care facilities, addressing gender disparities, and reducing costs associated with eye care treatments, potentially through advancement of the SSP.

## Introduction

Vision impairment affects 2.2 billion people globally, of which, around one billion cases are avoidable or yet to be addressed (World Health Organisation (WHO), 2019). In addition to being a global financial burden, unaddressed vision impairment affects many aspects of life, health, and sustainable development (Burton *et al*, 2021; United Nations (UN), 2018). A large proportion of those affected by unaddressed or preventable vision impairment live in low- and middle-income countries (WHO, 2019; Burton *et al*, 2021). Accessible eye care services can help to reduce the prevalence of avoidable blindness within a population by providing timely high-quality interventions to those who may benefit from them. Healthcare access has been defined as ‘the opportunity to reach and obtain appropriate health care services in situations of perceived need for care’ (Levesque, Harris and Russell, 2013). A recent scoping review (Malik *et al*, 2022) has identified a scarcity of high-quality evidence and the need for research surrounding the experiences of older age groups with eye care in Pakistan; specifically in view of an increasing prevalence of age-related eye conditions, significantly associated with more severe levels of impaired vision (Dineen et al, 2006; Jadoon *et al*, 2006).

Moreover, potential financial difficulties due to substantial health expenses are a global concern, especially in developing countries, where inadequacy of the state-provided health system results in high out-of-pocket (OOP) expenditure. Around 100 million people experience extreme poverty annually due to OOP expenditure on health services (World Health Organisation, 2022). In Pakistan, OOP payments finance 55% of total healthcare costs. The average annual household income per capita is 132,089 PKR (∼GBP481) in Pakistan, with the gap between the rich and poor widening (CEIC, 2019; Islam *et al*, 2022).

Considering global healthcare needs more broadly (i.e. beyond eye care), around half of the world’s population do not receive the healthcare they require (World Health Organisation, 2022). Universal health coverage (UHC) an initiative launched by the World Health Organisation is defined as, individuals having equal access to the health services they require, where and when they require them, and without financial hardship. Many low- and middle-income countries have implemented various health insurance schemes with a view to achieving UHC while reducing OOP costs (Bitran, 2014; Bredenkamp *et al*, 2015).

As part of recent and ongoing initiatives to achieve UHC in Pakistan by 2030, the Sehat Sahulat programme (SSP), a health insurance scheme, was launched in the Khyber Pakhtunkhwa province (KPK) in 2015 (Cheema *et al*, 2020). Initially users of the scheme had to apply for the ‘Sehat Insaf card’, which needed to be shown upon use at eligible hospitals. Currently, the SSP is available to all permanent residents in KPK, Punjab, Gilgit–Baltistan, Azad Jammu and Kashmir, Islamabad, district Tharpakar (Sindh), and to all transgender communities across Pakistan who are registered with the National Database and Registration Authority (NADRA) and have a computerized national identity card (CINC) (Sehat Sahulat Programme, 2022).

The SSP provides an allowance to families to use towards healthcare costs, which is renewed annually without rollover. The SSP can be used towards secondary care in-patient medical services with additional financial limits assigned for certain treatments (i.e. cancer, cardiological interventions, dialysis, and renal transplant), emergency situations, and in the event of maternity. Transport costs up to 1,000 PKR (GBP3.64) to reach a secondary health care facility are covered three times per year, and transport for patients referred by a secondary care hospital to a tertiary care hospital are also covered under the SSP (Din *et al.*, 2022). In the KPK province the ‘Sehat card plus’ initiative is being rolled out where higher allowances are provided for secondary and tertiary in-patient care (Health Department Khyber Pakhtunkhwa, 2021). The SSP is a cashless scheme for insured people, administered through the State Life Insurance Corporation (SLIC). The provincial government currently pays a fixed premium per eligible family to SLIC, which manages members’ in-patient healthcare expenses at participating private and public healthcare facilities. At the end of the 3-year contract period with SLIC, the provincial government receives a refund of 90% of any unspent net premium (Morgan and International Labour Organisation, 2019; Forman *et al.*, 2022; Shaikh & Ali, 2023). There is a paucity of research on the SSP’s use towards eye care services. To improve efficiencies in decision-making processes and resource allocation in healthcare, information regarding the SSP with respect to eye care services needs to be generated, which will in turn provide valuable evidence to both care providers and users, as the programme continues to gain popularity.

To capture comprehensive qualitative and quantitative evidence and to allow for a meaningful synthesis of the data the study adopted a mixed methods design to evaluate access of eye care services in Pakistan, with a focus on older age groups. The aim of this research is to answer the following research questions 1) What barriers do older age groups experience in relation to accessing eye care in Pakistan? 2) Is access to eye care influenced by the SSP and what other costs are associated with eye care access?

## Methods

### Study design and participants

**Figure 1.**
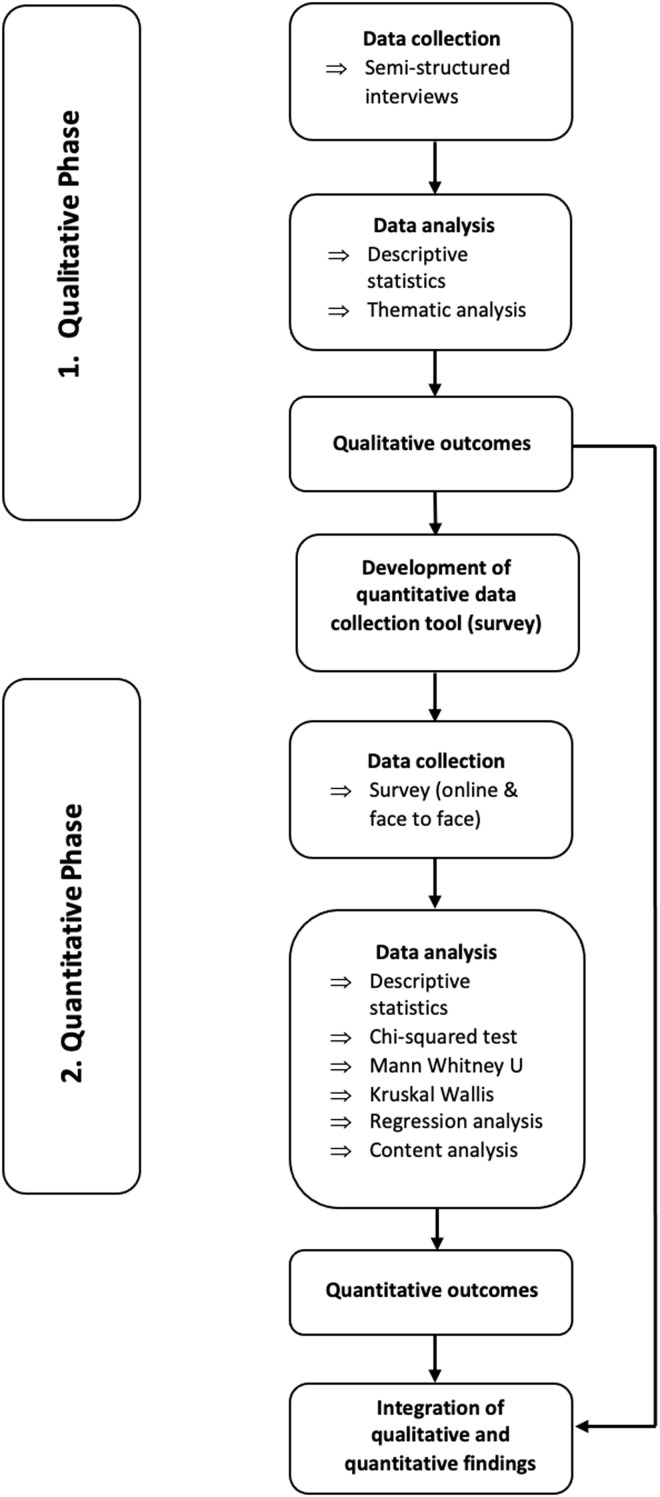
Illustration of exploratory sequential design of mixed methods study, showing initial qualitative phase, followed by survey development and secondary quantitative phase and ending with integration of outcomes from both phases.

To develop a detailed understanding of patient views and priorities in terms of eye care in Pakistan, an exploratory sequential mixed methods design was used (Fetters, Curry and Creswell, 2013) – see Figure 1. The design begins by exploring with qualitative interviews, to better understand the views, beliefs, and perspectives of participants towards access to eye care in Pakistan (phase one). Phase two involves building on the results of phase one, to design and use a survey tool tailored to meet the needs of the individuals being surveyed; allowing for the quantification of barriers to access eye care. Data from both phases were then integrated in a mixed methods display (Fetters, Curry and Creswell, 2013). Ethical approval for phase one of the study was granted by the ethics review board of the authors’ institute on the 1st of July 2021, and approval for phase two was granted on the 12th of November 2021.

An information sheet was provided to all participants recruited for the study. For the qualitative interviews, a consent sheet was emailed to participants to fill and return. For participants unable to sign and return the consent form, due to technological difficulties, verbal consent was obtained prior to starting the interview. For the online survey, participants provided implied consent by proceeding with the survey, as indicated on the first page: *‘By continuing with the survey, you are providing consent to participation’.* For participants recruited for the face-to-face survey consent was obtained prior to asking the survey questions, by asking the following statement: *‘Please tick the box next to this statement to confirm that you have read and understood the information sheet and agree to participate in this survey’.*

Purposive sampling was adopted in this study as participants were identified through personal and professional contacts and by word of mouth (Hibberts *et al.*, 2012). Participants aged 45 years and older were invited to take part in the study. The inclusion of participants aged 45 years and older aligns with the study’s focus on the older population, as age-related eye conditions (i.e. presbyopia) often begin to emerge in mid-life. With Pakistan’s rising life expectancy and aging population, the demand for eye care services is expected to increase (Dineen *et al.*, 2007; Najam & Bari, 2017). Addressing eye care needs from mid-life onward will help manage the growing demand and contribute to reducing avoidable blindness amongst the older population. An information sheet was given to all participants and consent obtained prior to involving them in the study. Detailed inclusion criteria for phase one and two can be found in Table 1.

**Table 1.** Inclusion criteria for both phases of exploratory sequential mixed methods study

**Phase 1: Qualitative interviews**	
Inclusion criteria	• Aged 45 years and older with experience of accessing the eye care system • Access to phone or device with internet connection • Able to communicate in either Urdu, Punjabi or English • Ability to give consent
**Phase 2: Online survey**	
Inclusion criteria	• Aged 45 years and older with experience of accessing the eye care system • Access to a device with internet connection • Able to read Urdu script • Ability to give consent
**Phase 2: Face–to-face survey**	
Inclusion criteria	• Aged 45 years and older • Presenting at one of the two hospital sites in Pakistan • Able to communicate in either Urdu, Punjabi or English • Ability to give consent

### Qualitative phase

#### Data collection

Data was collected through semi-structured interviews, with questions designed initially in English to meet the objectives of the study and based around the five abilities of the population which influence access (Levesque, Harris and Russell, 2013) (supplementary file 1). Interview questions were translated from English into Urdu and pre-tested with Urdu-speaking Pakistani nationals, which allowed us to test the suitability of questions and preferred language translations. Interviews were conducted during the COVID-19 pandemic, therefore, following the initial testing stage, telephone interviews were carried out as participants indicated a preference to this method of contact due to unreliable internet connections, which made online interviews problematic.

All interviews were carried out by the primary researcher with a second observer present to allow for accurate translation from English into Urdu and Punjabi (and vice versa), and to minimize any potential researcher bias. Interviews ranged from 10 to 30 minutes in length. The interviews took place from 14^th^ of July to 6^th^ of September 2021. After 11 interviews, saturation was reached. To ensure no relevant information had been missed two additional interviews were conducted, which confirmed that no new information could be obtained, resulting in a total of 13 remote interviews conducted. Interview transcripts were audio recorded, and translated, and transcribed into English.

#### Data analysis

Thematic analysis was used to analyse the qualitative interview data (Saldana, 2016). Line by line hand coding of each interview transcript was undertaken inductively by the primary researcher. This approach allowed for data familiarization and for data quality to be assessed by reviewing the variety of responses and elaboration of answers. An iterative process was adopted where codes were refined and updated as more interviews were coded. Codes were then combined to produce categories. Themes and sub-themes were then produced.

### Quantitative phase

#### Survey design

The survey was designed based on the outcomes from phase one of the study (supplementary file 2). The online survey consisted of 45 questions, beginning with closed-ended questions to obtain demographic information from participants. This was followed by questions (some of which adopted a Likert scale) exploring the participants’ knowledge of eye health, journey to the eye care provider, cost of eye care, and finally the participants’ overall experience with eye care services was explored, ending with an open-ended question and free text box for the participant to add any additional comments regarding their experience with eye care services in Pakistan.

Maintaining the overall data capture capabilities of the survey, a very slightly modified version of the survey was used for individuals who presented in-person to one of the two hospital survey sites. The slight modification was necessary to reflect the in-person nature of the data collection. The survey was split into two parts to better capture participants’ experiences with the facility they were presently visiting as well as previous eye care experiences. To ensure consistency and reliability of the two survey versions, both designs were piloted amongst five Pakistani nationals (these results were not included in the final dataset or analysis). This stage proved useful as it allowed for minor adjustments to the wording of some questions.

#### Data collection

A version of the survey was made available online, in Urdu script, for the general public in Pakistan to access from 5^th^ November 2021 to 30^th^ March 2022. Survey data was collected and managed using Research Electronic Data Capture (REDCap) tools (Harris *et al*, 2009; Harris *et al*, 2019). Accessing the survey online required participants to have access to a device with an internet connection. The participants also needed to be literate. To ensure widest possible participation, we identified two eye hospitals in Pakistan where hard copies of the survey were handed out. Interviewers were at hand locally to conduct the surveys face-to-face with illiterate participants. These two sites included: Al-Shifa Trust Eye Hospital, Rawalpindi, and a government hospital in Chakwal.

#### Sample size

Sample sizes were determined based on expected heterogeneity of population groups at selected survey sites (Hibberts *et al.*, 2012). Al-Shifa Trust Eye Hospital is one of the largest eye hospitals in the country, with some participants, from phase one, reporting travelling up to four hours to reach this hospital. We can therefore assume heterogeneity amongst the population will be higher for this location, and a sample size of 30 participants was set as a baseline. In contrast, the government hospital serves local populations in neighbouring villages with a relatively homogenous population, requiring a smaller sample, which was set at 15 participants. The surveys were conducted during the COVID-19 pandemic and therefore due to decreased activities at the hospitals, these sample sizes were deemed reasonable. The online dissemination of the survey aimed to target a variety of participants across Pakistan; therefore, a sample size of 100 participants was set.

#### Data analysis and statistical methods

Using outcomes of our earlier work and to allow for a comprehensive exploration of the relationships between variables, assumptions were formulated to identify relationships between variables and outcomes as suggested by the qualitative interviews (phase one) and literature (Malik *et al.*, 2022). Supplementary file 3 provides the assumptions and their outcomes. Each assumption was tested through some form of robust statistical analysis.

The survey data was analysed through IBM SPSS Statistics (Version 26). Categorical variables were summarized as frequencies and percentages, adjusting if respondents had left an item blank. Continuous variables were presented as mean ± SD, median, and range. The Shapiro–Wilk test of normality was also conducted for continuous variables. Chi-squared tests, Mann–Whitney U tests, and the Kruskal–Wallis tests were employed. All factors (from chi-square test) found to be significant (p values <0.05) were included in a regression model (binary/multinomial), as they were more likely to add to the improvement in prediction of the logits (Smith, 2018; Kwak and Clayton-Matthews, 2002; Chatterjee and Hadi, 2012). All independent variables were entered simultaneously (forced entry approach), and the model fit was assessed. All regression models presented in the study, demonstrated a good model fit through assessment of chi-square values (p-value <0.05) and the Hosmer–Lemeshow test (p-value ≥0.05) (Field, 2018; Laerd Statistics, 2017). Content analysis was used to analyse written data generated from the free text box at the end of the survey. An inductive approach to coding was utilized, which allowed for open coding (Elo and Kyngäs, 2008). Codes describing similar concepts were then aggregated to form categories.

### Integration of qualitative and quantitative outcomes

The integration of both qualitative and quantitative data occurred initially when qualitative outcomes from phase one were built upon by the development of a survey tool for phase two, and again at the reporting level through a joint display. In order to determine coherence between qualitative and quantitative findings the fit of data integration was assessed, resulting in four possible outcomes 1) *Confirmation:* findings from both phases agree with one another 2) *Complementarity:* findings from both phases show different, non-conflicting conclusions 3) *Expansion:* findings from both phases expand insights of the topic of interest (by addressing different aspects of the same topic or describing complementary aspects of the same topic) 4) *Discordance:* findings from both phases conflict/disagree with one another (Fetters, Curry & Creswell, 2013; Nollett *et al.*, 2019).

## Results

### Demographic factors

Thirteen participants took part in phase one (qualitative interviews) and had a mean age of 60 years old (± 10) with 54% male. The majority (9/13) resided in the Punjab province, with 2/13 from Sindh, and 1/13 in both KPK and the federal capital territory, with 54% reporting that they lived in a rural area. In (quantitative) phase two, a total of 163 participants responded to the survey, either taking part online or in-person at one of the two physical study sites. Of these, 58% were female and lived predominantly in urban areas (82%). Most participants resided in the province of Punjab (62%). A breakdown of participant demographic factors for each of the three survey sites is provided in Table 2. Full survey results can be found in supplementary file 4.

**Table 2.** Demographic factors for each of the three survey sites

Demographic Factors	n (%)			
	Online	Al-Shifa Trust Eye Hospital	THQ Hospital in Chakwal	Total
**Age group (years)**				
45–50	45 (38.1)	10 (33.3)	5 (33.3)	60 (36.8)
51–55	25 (21.2)	4 (13.3)	3 (20.0)	32 (19.6)
56–60	20 (16.9)	3 (10.0)	2 (13.4)	25 (15.3)
61–65	12 (10.2)	7 (23.3)	1 (6.7)	20 (12.3)
66–70	7 (5.9)	1 (3.3)	1 (6.7)	9 (5.5)
71–75	5 (4.2)	4 (13.3)	2 (13.3)	11 (6.7)
>76		1 (3.3)	1 (6.7)	2 (1.2)
Missing	4 (3.4)			4 (2.5)
**Gender**				
Male	75 (63.6)	11 (36.7)	9 (60.0)	95 (58.3)
Female	41 (34.7)	19 (63.3)	6 (40.0)	66 (40.5)
Prefer not to say/missing	2 (1.7)			2 (1.2)
**Province**				
Punjab	60 (50.8)	26 (86.7)	15 (100.0)	101 (62.0)
KPK	20 (16.9)	1 (3.3)		21 (12.9)
Federal Zone	16 (13.6)			16 (9.8)
Sindh	12 (10.2)			12 (7.4)
Azad Kashmir	6 (5.1)	3 (10.0)		9 (5.5)
Balochistan	3 (2.5)			3 (1.8)
Missing	1 (0.8)			1 (0.6)
**Community Setting**				
Urban	104 (88.1)	28 (93.3)	1 (6.7)	133 (81.6)
Rural	14 (11.9)	2 (6.7)	14 (93.3)	30 (18.4)
**Education**				
Illiterate	0 (0)	8 (26.7)	9 (60.0)	17 (10.4)
< High school	9 (7.6)	10 (33.3)	4 (26.7)	23 (14.1)
High school	9 (7.6)	3 (10.0)	2 (13.3)	14 (8.6)
> High school	99 (83.9)	9 (30.0)		108 (66.3)
Missing	1 (0.8)			1 (0.6)
**Employment**				
Full-time employee	42 (35.6)	5 (16.7)	1 (6.7)	48 (29.4)
Self-employed	28 (23.7)	1 (3.3)	5 (33.3)	34 (20.9)
Unemployed	11 (9.3)	21 (70.0)	2 (13.3)	34 (20.9)
Retired	16 (13.6)	3 (10.0)	1 (6.7)	20 (12.3)
Unable to work	6 (5.1)		6 (40.0)	12 (7.4)
Part-time employee	6 (5.1)			6 (3.7)
Looking for work	5 (4.2)			5 (3.1)
Missing	4 (3.4)			4 (2.5)

### Qualitative results

Following analysis of the thirteen interviews in phase one, a total of five themes emerged along with eleven sub-themes. The first three themes (i) ability to perceive; (ii) ability to reach; and (iii) ability to pay are based on Levesque, Harris and Russell, (2013) demand side abilities affecting accessibility. The fourth theme suitability of eye care service explores the quality of eye care services, patient satisfaction and patients trust in eye care providers, and the fifth theme reports on areas needing improvement as reported by the participants. These themes and sub-themes helped to formulate corresponding survey items used in phase two (Table 3).

**Table 3.** Joint display of qualitative and quantitative data. Emerging themes and sub-themes with overarching outcomes and sample quotes from phase one are presented alongside corresponding survey items, significant results from logistic regression/Mann–Whitney U tests and an assessment of fit of data integration

Themes from qualitative interviews	Qualitative outcomes	Sample quote(s)	Corresponding survey item(s)	Quantitative outcomes	Fit of data integration
**Ability to perceive**					
Eye health literacy	Illiterate people in rural areas have poor eye health literacy. The majority of participants were aware of cataract and diabetic retinopathy.	*‘I live in a village, here people don’t even know anything about the eyes, they don’t know what treatment is. There are very little educated people. So, because of this reason…people they get a lot of cataracts, some people struggle to see, and some people can’t see after it gets dark’.* (59-year-old, male, rural area)	Please select what eye related issues you are aware of from the following list: cataract, glaucoma, eye problems caused by diabetes, macular degeneration, dry eye, refractive error.	Awareness was highest for refractive error (59.5%), closely followed by cataract (54.0%). Those who were educated to a level greater than high school, were 0.16 times (CI 0.07–0.40, p = <0.001) more likely to be unaware of cataracts than those educated to a level less than high school.	Expansion
Perception of needs and desire for care	Majority of participants did not attend regular eye examination. Reasons for not attending regular eye checks included illiteracy, cost of eye appointment and lack of trust in eye care providers.	*‘Most people will only get their eyes checked if they have a problem, they won’t go for regular checks. To go to get checked by the doctor and pay them fees if they are not having issues no one goes’.* (60-year-old, male, rural area) *‘I myself get checked every 6 months, but most people don’t take interest and don’t get their eyes checked, but educated people take interest and go to get their eyes checked’.* (60-year-old female, urban area)	Have you had your eyes checked before? Select a reason for not getting your eyes checked. How long ago did you have your eyes checked? What reason(s) prompted you to get an eye test? How long do you wait between one eye check to the next? How often do you believe you should get your eyes checked? If you haven’t had an eye check in the last 2 years, please indicate why not.	Participants in the 51-to-60-year age category were 4.21 times (CI 1.50–11.82, p = 0.006) more likely to have their eyes tested previously than those in the below 50-year age category. Those who had studied to a level greater than high school were 5.62 times (CI 2.18 –14.47, p = <0.001) more likely than those who had studied to a level less than high school to have their eyes checked previously.	Confirmation
**Ability to reach**					
Availability of eye care services	Lack of government sector eye care services available in rural areas. Government services are preferred by those from lower socio-economic backgrounds.	*‘There aren’t any in our village, but in the next city … there is a rural health centre. But for the eyes there are no tests, treatments, or doctors there for it’.* (56-year-old male, rural)	Where do you get your eyes checked?	The private sector was the most popular (57.9%) to seek eye care from, followed by government (25.6%). Participants with high school level education were 6.6 times (CI 2.03 – 21.43, p = 0.002) more likely to seek care from a government service than private service, and 11.4 times (CI 2.44 – 53.18, p = 0.002) more likely to seek care from a charity service than private service, when compared to participants educated to a level > high school. Those who were unemployed were also 5.58 times (CI 1.05–29.69, p = 0.044) more likely to use a charity service than private service when compared to those who were employed.	Expansion
Transport	Long travel times to reach eye care provider for those living in rural areas.	*‘It is difficult because it (private eye clinic) is 25km from here and the roads are not in good condition. So, it is difficult because it takes up a whole day’.* (56-year-old, male, rural area)	How long does it take you to reach this eye care service? What mode of transportation do you use to reach this eye care service?	When asked how long journey times were to reach an eye care provider, the most common answer was 15 to 30 minutes (34.7%). When looking at the three survey sites independently, participants who visited Al-Shifa Trust Eye Hospital travelled the longest with 56.7% travelling more than 1 hour. Car was the most common mode of transport (62.6%), followed by bus (13.8%).	Expansion
Social support	Dependency on support to reach eye care service by all participants over the age of 60 years old.	*‘To travel in Pakistan is not easy, but it depends on who we are living with and if they have resources like a car and then they can drive you’.* (72-year-old, female, urban area)	Do you have any mobility issues? Do you rely on someone to take you to this eye care service?	Females were 2.54 times (CI 1.05 – 6.16, p = 0.039) more likely to rely on someone to reach an eye care facility than males. Females were also more likely to have a mobility issue than males amongst our sample (p = 0.027, chi-square test). Most participants (70%) who visited Al-Shifa Trust Eye Hospital relied on someone to help them reach the eye care facility despite only 13.3% reporting mobility issues.	Expansion
**Ability to pay**					
Appointment costs	The clinician who undertook the examination and the area in which the eye care facility was located influenced the amount charged for an eye test. Appointment fees were reasonable and affordable.	*‘The price for a regular check-up depends on the doctor’s facilities, if it’s a very good well-known doctor they charge more’.* (60-year-old, female, urban area) *‘The doctors’ fees were not that much it was between 1500-1800PKR’.* (48-year-old, male, urban area) *‘The first time we went to the doctor they took 400PKR. Then after when we went this time they took 300PKR. So, they usually take around this much so they don’t take a lot for checking the eyes’.* (60-year-old, female, urban area)	Estimate the total amount in PKR that you paid towards your last eye check-up. Estimate the total amount in PKR that you have ever paid towards eye care treatment. Who pays for your eye appointment/treatments? Who pays for your travel costs to reach an eye care facility? Where did you use money from to cover these costs? Did you find the eye care service you used affordable? Please rate its affordability on a scale from 1-10 with 1 being not affordable and 10 being very affordable.	The eye exam cost at the THQ hospital in Chakwal (mean = 23.33±90.37PKR, median = 0, range = 0-350, Shapiro–Wilk p < 0.001, n = 15) was less than a tenth of the costs of an eye exam at Al-Shifa Trust Eye Hospital (mean = 305± 382 PKR, median = 400PKR, range = 0-15000PKR, Shapiro–Wilk p = <0.001, n = 30), this difference was statistically significant (p = 0.001, Mann–Whitney U). The Mann–Whitney U test also showed a significant difference between where an eye care professional was trained and the cost of an eye test (p =0.010) with a higher appointment cost for those being examined by a professional trained in a high-income country (mean = 2364.29PKR, median=2000PKR, range=100-5900PKR, Shapiro–Wilk p=0.065, n=14) compared to locally trained in Pakistan (mean=1515.28PKR, median=650PKR, range=0-15000PKR, Shapiro–Wilk p = <0.001, n = 40). A statistically significant difference was found between community setting and the cost of an eye appointment (p = 0.006) with a higher eye appointment fee in urban areas (mean = 1746.51PKR, median =1500PKR, range= 0-15000PKR, Shapiro–Wilk p = <0.001, n = 77) when compared with rural areas (mean=626.67PKR, median =1000PKR, range= 0-3000PKR, Shapiro–Wilk p = 0.001, n = 15). Over half of the participants reported that they covered eye appointment, treatment, and travel costs themselves. When asked where the money they used to cover these costs came from, the majority (85.5%) said their income. Participants were asked to rate the affordability of the eye care service they visited on a scale from one to ten, with ‘1’ indicating not affordable and ‘10’ very affordable, the median score was 6 with a range of 1 to 10 (n =108) showing that the majority found services to be affordable.	Confirmation
Treatment costs	Variable treatment costs	*‘It costs a lot because I had to spend a lot of money, they used to call me in for check-ups every 2 weeks and then monthly. And I was taking medicine alongside. Approximately I spent 100,000 PKR. Because the medicine I was using for 1 year, and I kept getting additional tests’.* (48-year-old, male, urban area)	Estimate the amount you spent on eye care treatment.	Amount spent on eye care treatment: n = 72, Mean (±SD)= 14668.75 ± 40181.18 PKR, median = 4750 PKR, range = 0-300,000PKR, Shapiro–Wilk p = <0.001.	Confirmation
Micro health insurance	Awareness of the SSP micro health insurance programme not universal. Confusion was reported relating to the card’s availability, with many participants unsure if they were eligible to enrol or how to enrol for the health card. Some participants reported the use of the health card being used to cover the cost of eye care treatment	*‘Yes, I’ve heard about this government card. Yes, but things are very much distant… I have heard in a few cases that they are taking it for eye care treatment’.* (60-year-old, female, urban) *‘In our area the population is around 200,000 and out of that I think around 6% of people will have the card, the rest of the people are still waiting’.* (60-year-old, male, rural area)	Have you heard of the Sehat Sahulat programme? Are you eligible to enrol for the Sehat insaf card? Have you enrolled to receive a Sehat insaf card? On a scale from 1-10 with 10 being most difficult and 1 being easy, please select how you found enrolling onto the Sehat Sahulat programme. Have you used the card towards eye care treatment? Would you have still accessed eye care and sought eye related treatment if the Sehat health card was not available to you? Since receiving the card on a scale from 1 – 10 how much more likely are you to go get your eyes checked with 1 being not likely and 10 being very likely.	There was no significant difference between the five demographic variables (age p = 0.970, gender p = 0.081, education level p = 0.096, community setting p = 0.656 and employment status p = 0.922) and whether one had heard of the SSP, with 63.5% of participants (n = 159) aware of the SSP. Only 49 participants were eligible to enrol to receive the health card. Females were 3.08 times (CI 1.31-7.25, p = 0.010) more likely to not be eligible to enrol for the Sehat Insaf card when compared to males. Out of those eligible to enrol, 18 participants had undertaken enrolment. Seven participants had received their health card, yet only one person had used their card towards eye care treatment with no participants using the card towards travel costs to reach a medical facility.	Confirmation
**Suitability of eye care service**					
Quality of eye care facilities	Views regarding the quality of government and private sectors differed between and within each sector.	*‘The government hospitals have very good facilities, very good operations are carried out and good services for children and older people. They do all types of operations and they are very good’.* (60-year-old, female, urban area)	Which sector has better quality equipment and facilities; government or private?	Participants were asked which sector has better quality equipment and facilities. The private sector scored slightly higher than government with 59.0% of participants stating equipment was better in private clinical settings.	Confirmation
Trust in eye care providers	Lack of trust in eye care providers as prioritising profit over patient care. Increased trust in eye care professionals who were trained abroad in developed countries. These professionals were based in private eye hospitals.	*‘The doctors…in the government hospitals tell you to go to their private clinics where they will charge you but give you proper treatment in return. So, from the government hospitals they take you to the other side just to make money’.* (59-year-old, male, rural area) *‘I found that experience was very good, and the doctor … was very patient… He took education from America he said’.* (72-year-old, female, urban area)	How satisfied were you with the eye care professional that checked your eyes? Where was the eye care professional that checked your eyes trained?	Out of 121 participants 38.0% reported that they were very satisfied with the eye care professional that checked their eyes at their last visit, 47.9% were somewhat satisfied, 9.9% were neither satisfied or dissatisfied and 4.1% were not satisfied. 23/122 (18.9%) of participants reported that the eye care professional that tested their eyes was trained in the UK/USA. There was no significant difference between where an eye care professional was trained and the sector they worked in (private/government) (p = 0.116, chi-square).	Complementarity
Patient satisfaction	Long wait times affecting patients’ satisfaction with eye care experience. Long wait times as a result of queue system. Shorter wait times in private clinics.	*‘You have to go very quickly early morning, if the hospital opens at 9am, people will be there for 6am…You have to wait in a very long queue until your number is called. And if that day your number doesn’t get called and the doctors’ day finishes, for example their shift is from 9am -1pm, and it reaches 1 pm, the doctor will leave without checking you. Then you have to go back again the next day. Then you have to go through this procedure again… and again if it doesn’t get called you need to go the third day, this is quite difficult’.* (54-year-old, female, urban)	How long did you have to wait in the eye care facility before getting seen? Overall, how satisfied were you with your overall experience with this eye care service? Did you book your eye appointment in advance, or did you have to wait in a queue system on the day?	There was no significant difference between wait time and whether a government or private service was used (p = 0.539), whether a queue system was used or booking in advance (p = 0.372) or the overall level of satisfaction with the eye care service (p = 0.102). Satisfaction levels with the eye care professional that checked the participants’ eyes and with their overall experience with a previous eye care service were generally high.	Complementarity
**Participant reported areas for improvement**	Five participant reported areas for improvement relating to eye care services in Pakistan were identified	*‘In the small cities more services are needed, like the facilities in the district should be made available in these areas especially the smaller populated cities attached to them. The city near us there are a lot of villages around it. So, if in these cities the government set up a permanent eye camp or give facilities in a hospital that would be even better’.* (56-year-old, male, rural area)	Out of the following options, please rank them from 1 to 5 based on their importance in improving current eye care services, with 1 being the most important and 5 being the least important. • Increasing the availability of eye care services in rural areas • Free eye care treatment • Free transport to eye care services • Shorter wait times in eye care facilities • More lifts and ramps in eye care facilities	Ranked most important to least (mean ± SD): • Increasing the availability of eye care services in rural areas (1.71 ± 0.82) • More lifts and ramps in eye care facilities (2.10 ± 0.91) • Free eye care treatment (2.39 ±0.88) • Shorter wait times in eye care facilities (4.32 ± 0.79) • Free transport to eye care services (4.45 ± 0.72)	Expansion

### Content analysis

Thirty-four participants utilized the free text box. The resulting five categories identified through content analysis were: positive eye care experience, suggested changes to eye care services, eye health literacy, SSP, and eye care sector (Table 4). The most cited category was positive eye care experience, participants noted that they were satisfied with their eye care experience, however no specific details were shared as to what aspects of their eye care journey created this positive outcome. Within the category ‘suggested changes to eye care services,’ four participants commented on the need for free examinations and/or treatment. Three participants described long wait times, with one stating that long wait times were the reason they did not return for another eye exam. Three participants also commented on eye care facilities needing upgrades. While two participants discussed the need for more well-trained staff at eye care facilities and a further two participants commented on the need for nearby, easily accessible eye care services. Four participants provided comments demonstrating their understanding of the importance of eye health. The SSP also received comments detailing confusion surrounding eligibility for the programme, enrolling to receive a health card, and utilization of the health card. Comments relating to eye care sector were made by two participants, with one detailing that the equipment in government hospitals seems good but they are unsure if they are ‘properly used and maintained’.

**Table 4.** Frequency of categories and sub-categories identified through content analysis of participants additional comments on experience with local eye care services

Categories	n
Positive eye care experience	15
Suggested changes to eye care services	14
*Cost*	4
*Wait times*	3
*General upgrade*	3
*Workforce*	2
*Accessibility*	2
Eye health literacy	4
Sehat Sahulat programme	4
*Eligibility*	2
*Enrolment*	1
*Utilisation*	1
Eye care sector	2

## Discussion

This mixed methods study has explored the experiences of people aged 45 years and older, with accessing eye care services in Pakistan. Overall, this study provides detailed evidence and novel insight from users of eye care. The outcomes of our study confirm that considerable barriers exist including illiteracy, long travel times, female gender, older age, mobility issues, and cost all limiting access to eye care in Pakistan. Awareness surrounding use of the SSP was poor, with the programme seldom used towards eye care costs.

The first barrier identified related to a person’s eye health literacy level. Previous literature also identified poor eye health literacy as a barrier to accessing eye care (see Safi *et al.*, 2018; Tatham, Weinreb & Medeiros, 2014; Cassum *et al.*, 2020; Jalal & Younis, 2014; Itrat *et al.*, 2007; Qureshi, 2012; Ali *et al.*, 2022). Within our sample many participants believed that if they were asymptomatic, there was no reason to schedule an eye exam. This highlights the lack of knowledge surrounding the importance of routine eye examinations and generally asymptomatic eye conditions in their early stages, such as glaucoma and diabetic retinopathy. For both conditions, it has been shown that routine asymptomatic examinations allow for earlier detection and treatment, thus reducing the likelihood and impact of visual impairment (Safi *et al.*, 2018; Tatham, Weinreb & Medeiros, 2014).

The next major barrier, impacting access to eye care, was the participant’s ability to reach an eye care provider. While this aspect is likely to vary and be dependent on geographical and socioeconomic attributes, it became evident that people living in rural areas in Pakistan struggled to travel long distances to reach secondary and tertiary eye care facilities in urban areas. Long travel distances for people living in rural areas to access eye care services have also been reported in the literature as a barrier to timely eye treatment in both Baluchistan and Sindh provinces (Jadoon *et al.*, 2007; Lane *et al.*, 2018). Transport was also an issue for those who could not drive and had to rely on others to take them to an eye care provider. Due to the strong family support system in Pakistan this duty would usually fall to a member of their family, requiring them to take time away from their work to provide support for their family member (Cassum *et al.*, 2020; Jalal & Younis, 2014; Itrat *et al.*, 2007; Qureshi, 2012). Females were also more likely to require support to reach an eye care facility. Gender discrimination is present in various aspects of Pakistani culture, as authority over family decisions and finances is usually given to male heads of household, limiting access to healthcare for females (Ali *et al.*, 2022; Anwar, Green & Norris, 2012) though, in phase two of our study, females were more likely to have mobility issues, which may have influenced this outcome.

Exploring participant views on eye care providers has helped improve understanding of the population’s perceptions of this workforce and how it may influence their decision on which eye care services to access. Participants preferred eye care providers who had received their training abroad because they felt they received a superior examination from such individuals due to the higher level of quality of training and knowledge associated with degrees obtained in developed countries. Some participants showed a lack of trust in eye care providers as they felt they prioritized generating a profit over the patient’s needs. This therefore negatively impacted their decision to seek eye care.

Another key and novel aim of this study was to investigate how access of eye care services is influenced by economic aspects including the SSP. When discussing cost as a barrier to access of eye care services, participants largely felt that services were affordable and they covered any associated costs themselves. However, cost of treatment, for example, for cataract surgery varied depending on the type of intraocular lens selected and the ophthalmologist carrying out the surgery and in some instances proved to be costly. It was observed that people from lower socio-economic backgrounds struggled to afford services provided by the private sector, and subsequently sought care from government and charity services. This suggests that cost remains a barrier to uptake of eye care services in Pakistan, with particular emphasis on cost of cataract surgery (Haider, Hussain & Limburg, 2003; Anjum *et al.*, 2006; Jadoon *et al.*, 2007; Lane *et al.*, 2018).

Findings from part one of the study found the overall influence of the SSP on eye care service utilization to be low. This was also confirmed in part two of the study as only one participant had used the Sehat Insaf card towards eye care treatment. Low utilization of the SSP with regard to general healthcare has also been reported in previous studies (Cheema *et al.*, 2020; Habib & Zaidi, 2021). A reason given for low utilization is that the Sehat health card can only be used at a select few facilities which meet a certain criterion, therefore people using the programme have to seek out facilities which accept it. Moreover, the SSP does not cover primary healthcare services or patients who visit outpatient departments (OPD). As a result, its use in ophthalmology departments is limited as most patients are managed in the OPD (McDonald & Iordanous, 2022). However, all in-patient services are covered by the SSP, allowing the SSP to be used for certain eye related surgeries (i.e. cataract, trabeculectomy, and so on) within ophthalmology departments (Morgan and International Labour Organisation, 2019).

The Sehat Insaf card can also be used to cover travel costs to reach a medical facility, yet no participants had claimed this. Although our survey did not investigate how long participants had held the card for, and as the scheme is relatively new, they may not have had an opportunity to utilize it to its full potential. Since dissemination of the surveys between November 2021 and March 2022, eligible health facilities can now assess patients under the SSP without them needing to enrol to receive the Sehat Insaf card (Sehat Sahulat Programme, 2022; Health Department Khyber Pakhtunkhwa, 2021; Hasan *et al.*, 2022). Therefore, it is possible that more patients may be inclined to utilize the SSP towards eye care as accessibility to the programme is increasing due to on-going roll out and wider spread eligibility.

With this study, we are hoping to provide useful insight into the issues people experience when accessing eye care. Listening to patients and service users is essential to enable decision makers and health departments to further improve clinical services and work towards achieving UHC. When asked to rank the five areas of improvement, participants ranked ‘increasing the availability of eye care services in rural areas,’ as their key priority. Healthcare planners are already working these aspects and Pakistan’s national health policy of 2001 recognized an urban bias in the health sector and aimed to address it by expanding public healthcare services to rural areas (Ministry of Health and Government of Pakistan, 2001). The responses our participants provided indicate that resolving access barriers is ongoing and requires a long-term view and continued funding efforts (Naz, Ghimire & Zainab, 2021).

## Strengths and limitations

The use of a mixed methods approach allowed for utilization of both qualitative and quantitative methods which allows for increased strength than each of these approaches alone (Creswell & Plano Clark, 2011). Part one identified a wide range of barriers, while part two demonstrated the magnitude of each barrier. The study also operationalized the framework to healthcare access by Levesque, Harris & Russell, (2013). The study focused on the demand side features of this model and also allowed for new themes to arise through inductive thematic analysis, thus providing comprehensive new evidence on eye care service use in Pakistan.

Non-random sampling techniques were used due to limited accessibility and time constraints (Etikan, Musa & Alkassim, 2016). Within our sample slight over-representation within the data set occurred, for example the majority of participants were educated to a level greater than high school. Individuals with a higher level of education are more likely to understand research activities and participate (Nirmalan *et al.*, 2004). We mitigated this by undertaking face-to-face surveys at the two hospital sites in the Punjab province, obtaining a more balanced sample and valuable data. The addition of face-to face surveys strengthened the validity and completeness of data collection as an interviewer was at hand to remove any ambiguities surrounding questions. It was also found that fewer questions were skipped in the face-to-face surveys when compared with the online survey. However, due to time constraints we were unable to check the interviewer’s inter-rater reliability. While providing novel evidence on barriers to eye care use, access, and costs, there remains considerable variability in geographic structure and socioeconomic capacity across Pakistan. Therefore, any generalising of the results to the whole country should be undertaken with caution. Furthermore, the SSP was not available at the government hospital in Chakwal at the time of the survey. Although the hospital provides free healthcare services to patients, this may have influenced their reliance on the SSP for eye care needs. This presents a limitation of the study, as the unavailability of the SSP at the facility could have impacted participants’ responses to questions related to the programme. Future research should consider comparing facilities that accept SSP with those that do not to gain deeper insights into the programme’s role in improving eye care accessibility.

Representation was achieved in all provinces apart from Gilgit–Baltistan, and the majority of responses were from the Punjab province despite best efforts to recruit more widely. However, the recruitment success is showing a similar distribution of results from provinces compared to other studies investigating health seeking behaviour in Pakistan (Naz, Ghimire & Zainab, 2021; Anwar, Green & Norris, 2012). Reasons given for this unequal distribution include more densely populated provinces including the Punjab and Sindh provinces and more healthcare facilities in urban areas. Additionally, Pakistan has a diverse range of cultures and languages, primarily in rural areas across all provinces (Naz, Ghimire & Zainab, 2021). This could also have been a contributing factor, as our online survey was only available in Urdu, the national language of Pakistan and used for formal written communication. Future research could consider offering the survey in regional languages, based on the location of survey dissemination, to ensure better comprehension and inclusivity.

Participants were given the option to skip questions they did not want to answer in the survey. In order to minimize participants skipping questions a number of pre-designed categories were available for most questions to encourage the participant to select an answer for each question. This approach was successful with a high response rate for such questions compared to a lower response rate where free text options were given. However, the use of these categories in some instances may have caused some unintentional, but unavoidable, bias onto participants, that is, when exploring participants eye health literacy and practices. Moreover, participants were asked to rank statements covering five areas for improvement, many misunderstood the question and rated each statement individually on a scale from one to five, resulting in a lower number of responses analysed (n = 31).

Due to the predominantly remote nature of data collection during the COVID-19 pandemic, detailed information on participants’ eye health, such as visual acuity and existing eye conditions, was not gathered. However, some participants voluntarily shared insights about their personal eye health during qualitative interviews, providing a glimpse into their knowledge and eye care-seeking behaviours. This suggests that access to eye care may be influenced by an individual’s specific eye health condition(s). Future research can aim to measure these factors for a more comprehensive understanding of participants’ eye care behaviours.

A final potential limitation is that we did not directly assess whether other health conditions impaired access to eye care facilities. For example, patients with dementia may experience limited access to eye care services due to increased social isolation or inappropriate care. It has been noted that certain health conditions including depression, dementia, cardiovascular disease, and lung cancer are associated with an increased risk of vision impairment (Burton *et al.*, 2021; Luo *et al.*, 2018; Mohile *et al.*, 2011; Zheng *et al.*, 2017; Crews *et al.*, 2017). Such conditions also have a higher prevalence amongst the elderly cohort (Crews *et al.*, 2017). Therefore, it is important that future research considers the effect of certain health conditions on access to eye care.

## Implications for practice

The burden of eye diseases in Pakistan is expected to rise as life expectancy rises and the population ages (Dineen *et al*., 2007; Najam & Bari, 2017). Inadequate access to eye care services will result in a larger backlog of people who need but do not receive eye care. The current study has identified and quantified barriers surrounding access to a variety of eye care services in Pakistan, covering all sectors and types of services from tertiary care eye hospitals in urban cities to eye camps in rural villages. This provides eye care service providers with information relating to barriers that need to be addressed, to improve accessibility of their services. By improving access to eye care services for older age groups, the eye care sector will be better positioned to manage the increasing demand on services due to age-related eye conditions and thus contribute to the reduction of avoidable blindness. Additionally, cost was identified as a barrier to accessing eye care in Pakistan. This study provides valuable insights into the low awareness and utilization rates surrounding the SSP and eye care. Further evaluations and improvements to the programme are critical in the effort to provide affordable eye care to all citizens in the country. Future research can focus on barriers and enablers to accessing eye care in rural locations across the country as the language and cultural norms vary between areas. Therefore, exploring these areas may allow for culturally sensitive targeted interventions to improve access, which may be better accepted by such communities.

## Conclusion

This mixed methods study has explored participants experiences accessing their local eye care services in Pakistan. Barriers to accessing eye care have been identified. Future efforts and initiatives should focus on continuing efforts to provide improvements to educating the public on eye health, increasing availability of secondary eye care services in rural areas, improving accessibility within eye care facilities, addressing gender disparities, and reducing costs associated with eye care treatments, potentially through advancement of the SSP. Most healthcare systems face challenges due to resources being scare, but despite these constraints, progress continues to be made.

## Supporting information

Malik et al. supplementary material 1 Malik et al. supplementary material

Malik et al. supplementary material 2 Malik et al. supplementary material

Malik et al. supplementary material 3 Malik et al. supplementary material

Malik et al. supplementary material 4 Malik et al. supplementary material

## Supplementary material

The supplementary material for this article can be found at https://doi.org/10.1017/S1463423625100261.
