# Supplementary material for: Barriers to accessing eye care in Pakistan: a mixed methods study
Source: Prim Health Care Res Dev. 2025 Jul 15;26:e58. doi: 10.1017/S1463423625100261 (PMC12260732; doi:10.1017/S1463423625100261)
Supplement: Malik et al. supplementary material 1 — Malik et al. supplementary material [file S1463423625100261sup001.docx]

Supplementary file 1

Semi-Structured Interview Questions

1. How old are you?
2. Which area are you from? (Name of district/tehsil, is this a city, town or village?)
3. What eye care services are available in your area? (Prompts: Teaching hospitals, eye hospitals, eye departments in DHQ or THQ hospitals, GP or ophthalmologist in BHU/RHC, private clinics, eye camps, Hakims)

**Ability to perceive**

1. What eye related problems do you know about? OR When I say eye problems what comes to your mind?
2. Do you have any current eye related issues, or any history of eye problems? (If answers yes continue to Q.6 if no skip to Q.11)
3. What are/were they?

**Ability to seek**

1. Did you go/have you gone anywhere to get your eye problem checked or to get treatment for it?
2. (Answers no) Why did you not seek treatment for your problem?
3. (Answers yes) How long after you became aware of the problem did you seek treatment? When did this problem occur? (approximate month/year)
4. Did you make the decision to seek treatment or did someone else decide on your behalf?
5. Do you go for regular eye checks even if you are not experiencing any problems?

**Ability to reach**

1. Where did you go with your problem? And/or Where do you go for your regular check and how often do you go?
2. How far did/do you need to travel to get your eyes checked?
3. How did/do you get there? (Walk, car, cycle, bus, train)
4. Did/do you go alone, or did/does someone accompany you?
5. Was/is it difficult or easy to take time out to go there?
6. Do you have any mobility issues? If so did/does this have any impact in your ability to reach the eye care service?

**Ability to pay**

1. Who pays for your eye care and travel costs?
2. Did/do you have to pay for the eye care service and/or any treatment? If so, how much? Did/do you use money from your income/savings or borrow money to cover the costs?
3. Have you heard of the Sehat Sahulat programme? (If answers no skip to Q.27)
4. Are you eligible to enrol for it? If so, have you enrolled to get a health card? (If answers no skip to Q.27)
5. Do you share use of the health card with other members of your family? If so, are anyone’s needs prioritised over another?
6. Have you or anyone in your household used the health card? Was it used on eye care treatment?
7. Has the programme changed the way you feel about regular health checks and seeking treatment for your health-related issues?
8. Have you used it to cover the cost of transportation to a medical facility?
9. Did you have to pay out of your pocket for any extra costs for the medical appointment, treatment or transportation to the eye care facility?

**Ability to Engage**

1. How involved were you in the decision making and treatment decisions regarding the health of your eyes?
2. How was the quality of the eye care service you used? OR Were you satisfied with the eye care service provided and the outcome?
3. What changes would you make to your experience to improve it for the next visit?
4. Is there anything else you want to tell us about your experience with eye care in Pakistan?
