# Supplementary material for: Barriers to accessing eye care in Pakistan: a mixed methods study
Source: Prim Health Care Res Dev. 2025 Jul 15;26:e58. doi: 10.1017/S1463423625100261 (PMC12260732; doi:10.1017/S1463423625100261)
Supplement: Malik et al. supplementary material 2 — Malik et al. supplementary material [file S1463423625100261sup002.docx]

Supplementary file 2

Online survey questions

**Background information about yourself**

1. Please select your age group from the list below.

45-50 years

51-55 years

56-60 years

61-65 years

66-70 years

71-75 years

>76 years

1. What gender are you?

Male

Female

Prefer not to say

1. What province do you live in?

Punjab

Sindh

Balochistan

Gilgit Baltistan

Azad Kashmir

KPK

Federal zone

1. Please select what type of area you live in.

Urban (i.e. city/town)

Rural (i.e. village/remote area)

1. Please select your highest level of education.

Less than high school

High school

More than high school

1. Please select your working status from the following list.

Full-time employee

Part-time employee (working less than 20 hours a week)

Retired

Self-employed

Looking for work

Choose not to work

Unable to work

**Knowledge surrounding eye health**

1. Please select what eye related issues you are aware of from the following list:

Cataract

Glaucoma

Eye problems caused by diabetes

Macular degeneration

Dry eye

Refractive error (causes blurred vision and requires treatment with prescription glasses)

1. Have you had your eyes checked before?

Yes

No (ask question 9 then end survey)

1. Please select a reason(s) for not getting your eyes checked from the following list.

Not having any eye related issues

Cannot afford it

Unable to travel to an eye care facility

Don’t have time

Don’t know where to go to get my eyes checked

My eyes are not a health priority right now

Other, please specify

1. How long ago did you have your eyes checked?

Less than 3 months

3-6 months

6 months – 1 year

1 year – 2 years

2-3 years

More than 3 years

1. From the following list, what reason(s) prompted you to get an eye test for yourself? (Can select more than one response)

Routine eye test

Change in level of vision

To purchase or repair/replace spectacles

Contact lens check

Headaches

Family history of eye disease

Glaucoma monitoring

Cataract monitoring

Diabetic retinopathy monitoring

Monitoring of age-related macular degeneration

Advised by other healthcare provider

Needed an emergency eye check

Other, please specify

1. How long do you wait between one eye check to the next?

1-3 months

3-6 months

6-12 months

1 year-2 years

2-3 years

More than 3 years

1. How often do you believe you should you get your eyes checked?

Every 1-3 months

Every 3-6 months

Every 6-12 months

Every 1 year-2 years

Every 2-3 years

More than 3 years

1. If you haven’t had an eye check in the last 2 years, please indicate why not:

Select all that apply.

Have not yet made my next appointment

Do not attend regular eye checks

Have not had a change in vision

Cannot afford to pay for a check or any subsequent treatment

Don’t have time

Cannot travel to an eye care provider

My eyes are not a health priority right now

Other, please specify

**Journey to your eye care provider**

1. Where do you get your eyes checked?

Government service

Private service

Charity service

Military service

Other, please specify

1. How long does it take you to reach this eye care service?

Less than 15minutes

15-30 minutes

30 minutes – 1hour

1-2 hours

2-3 hours

More than 3 hours

1. What mode of transportation do you use to reach this eye care service? (Can select more than one answer)

Motorcycle

Car

Rickshaw

Bus

Walk

1. Do you have any mobility issues?

Yes

No

1. Do you rely on someone to take you to this eye care service?

Yes

No

1. What is the most convenient day for you to get your eyes checked?

Monday – Friday

Weekends

Any day

1. What is the most convenient time for you to get your eyes checked?

Morning

Afternoon

Evening

Any time

**Cost of eye care**

1. Who pays for your eye appointment/treatments? (Can select more than one option)

Myself

Family

Health insurance

Other, please specify

1. Who pays for your travel costs to reach an eye care facility? (Can select more than one option)

Myself

Family

Health insurance

Other, please specify

1. (If patient selects myself for Q23 or 24 this question will also be asked) Where did you use money from to cover these costs:

Income

Savings

Borrowed

Other, please specify

1. Estimate the total amount in PKR that you paid towards your last eye check-up (appointment fee).

Rs.

1. Estimate the total amount in PKR that you have ever paid towards eye care treatment (i.e., cost of glasses/drops/medication/surgery).

Rs.

1. Estimate the total amount in PKR that you have spent on travel costs to reach an eye care facility.

Rs.

1. Did you find the eye care service you used affordable? Please rate its affordability on a scale from 1-10 with 1 being not affordable and 10 being very affordable.
2. Have you heard of the Sehat Sahulat programme?

Yes

No (skip to Q39)

1. Are you eligible to enrol for it?

Yes

No (skip to Q39)

1. Have you enrolled to receive a Sehat health card?

Yes

No (skip to Q39)

1. On a scale from 1-10 with 10 being most difficult and 1 being easy, please select how you found enrolling onto the Sehat Sahulat programme.
2. Have you received your health card?

Yes

No (skip to Q39)

1. Have you used the card towards eye care treatment?

Yes

No (continue to question 37)

1. Would you have still accessed eye care and sought eye related treatment if the Sehat health card was not available to you?

Yes

No

1. Have you used the card to cover the cost of transport to reach a medical facility?

Yes

Used for only part of the cost

No

1. Since receiving the card on a scale from 1 – 10 how much more likely are you to go get your eyes checked with 1 being not likely and 10 being very likely.

**Overall experience with eye care service**

1. Which sector has better quality equipment and facilities:

Government

Private

1. How satisfied were you with the eye care professional that checked your eyes?

Not satisfied

Neither dissatisfied or satisfied

Somewhat satisfied

Very satisfied

1. Was the eye care professional that checked your eyes trained in:

Pakistan

United Kingdom

United Sates of America

Other

Unsure

1. How long did you have to wait in the eye care facility before getting seen?

Less than 15minutes

15-30 minutes

30 minutes-1 hour

1-2 hours

2-3 hours

3-4 hours

More than 4 hours

1. Did you book your eye appointment in advance, or did you have to wait in a queue system on the day?

Booked in advance

Queue system for same day appointment

1. Overall, how satisfied were you with your overall experience with this eye care service?

Not satisfied

Neither dissatisfied or satisfied

Somewhat satisfied

Very satisfied

1. Out of the following options, please rank them from 1 to 5 based on their importance in improving current eye care services, with 1 being the most important and 5 being the least important.

Increasing the availability of eye care services in rural areas

Free eye care treatment

Free transport to eye care services

Shorter wait times in eye care facilities

More lifts and ramps in eye care facilities

1. Please use the box below to type any additional comments about your experience with your local eye care services.

Face to face survey questions

**A survey evaluating access and utilisation of eye care services amongst the elderly population in Pakistan**

Please tick the box next to this statement to confirm that you have read and understood the information sheet and agree to participate in this survey.

**Part 1**

**Background information about yourself**

1. Select your age group from the list below.

45-50 years

51-55 years

56-60 years

61-65 years

66-70 years

71-75 years

>76 years

1. What gender are you?

Male

Female

Prefer not to say

1. What province do you live in?

Punjab

Sindh

Balochistan

Gilgit Baltistan

Azad Kashmir

KPK

Federal zone

1. Select what type of area you live in.

Urban (i.e. city/town)

Rural (i.e. village/remote area)

1. Select your highest level of education.

Less than high school

High school

More than high school

None of these

1. Select your working status from the following list.

Full-time employee

Part-time employee (working less than 20 hours a week)

Retired

Self-employed

Looking for work

Unemployed

Unable to work

**Knowledge surrounding eye health**

1. Select what eye related issues you are aware of from the following list:

Cataract

Glaucoma

Eye problems caused by diabetes

Macular degeneration

Dry eye

Refractive error (causes blurred vision and requires treatment with prescription glasses)

1. Is this your first visit to this eye hospital or any other eye care facility?

Yes **(If your answer is yes, skip to part 2 of survey)**

No

1. How long ago did you have your eyes checked?

Less than 3 months

3-6 months

6 months – 1 year

1 year – 2 years

2-3 years

More than 3 years

1. How often do you believe you should you get your eyes checked?

Every 1-3 months

Every 3-6 months

Every 6-12 months

Every 1 year-2 years

Every 2-3 years

More than 3 years

1. How long do you wait between one eye check to the next?

1-3 months

3-6 months

6-12 months

1 year-2 years

2-3 years

More than 3 years

1. If you haven’t had an eye check in the last 2 years, please indicate why not:

Select all that apply.

Have not yet made my next appointment

Do not attend regular eye checks

Have not had a change in vision

Cannot afford to pay for a check or any subsequent treatment

Don’t have time

Cannot travel to an eye care provider

My eyes are not a health priority right now

Other, please specify

N/A I have had my eyes tested within the last 2 years

1. From the following list, what reason(s) prompted you to get an eye test for yourself today? (Select all that apply)

Routine eye test

Change in level of vision

Needed an emergency eye check

To purchase or repair/replace spectacles

Contact lens check

Headaches

Family history of eye disease

Glaucoma monitoring

Cataract monitoring

Diabetic retinopathy monitoring

Monitoring of age-related macular degeneration

Advised by other healthcare provider

Other, please specify

**Journey to your eye care provider**

1. Where did you go to get your eyes checked previously?

Government service

Private service

Charity service

Military service

Other, please specify

1. How long did it take you to reach this eye care service?

Less than 15minutes

15-30 minutes

30 minutes – 1hour

1-2 hours

2-3 hours

More than 3 hours

1. What mode of transportation did you use to reach this eye care service? (Can select more than one answer)

Motorcycle

Car

Rickshaw

Bus

Walk

1. Do you have any mobility issues?

Yes

No

1. Did you rely on someone to take you to this eye care service?

Yes

No

1. What is the most convenient day for you to get your eyes checked?

Monday – Friday

Weekends

Any day

1. What is the most convenient time for you to get your eyes checked?

Morning

Afternoon

Evening

Any time

If your last eye check **was not** done at the eye hospital you are visiting today, please also answer the following questions. If your last eye check **was** carried out at the hospital you are visiting today, **please skip to question 26**.

1. How long did it take you to reach the hospital today?

Less than 15minutes

15-30 minutes

30 minutes – 1hour

- 1. hours

2-3 hours

More than 3 hours

1. What mode of transportation did you use to get to the eye hospital today? (Can select more than one answer)

Motorcycle

Car

Rickshaw

Bus

Walk

1. Did you rely on someone to take you to bring you here today?

Yes

No

1. How much did you pay for your eye check-up today (appointment fee)

Rs.

1. How much did it cost you to travel to the eye hospital today?

Rs.

**Cost of eye care**

1. Who pays for your eye appointment/treatments? (Can select more than one option)

Myself (also answer Q28)

Family

Health insurance

Other, please specify

1. Who pays for your travel costs to reach an eye care facility? (Can select more than one option)

Myself (Also answer Q28)

Family

Health insurance

v

Other, please specify

1. If you answered myself for the last two questions please answer this additional question, otherwise skip to question 29.

Where did you use money from to cover these costs?

Income

Savings

Borrowed

Other, please specify

1. Estimate the total amount in PKR that you paid towards your last eye check-up (appointment fee).

Rs.

1. Estimate the total amount in PKR that you have ever paid towards eye care treatment (i.e., cost of glasses/drops/medication/surgery).

Rs.

1. Estimate the total amount in PKR that you have spent on travel costs to reach an eye care facility.

Rs.

1. Did you find the eye care service you used affordable? Please rate its affordability on a scale from 1-10 with 1 being not affordable and 10 being very affordable.

Enter number:

1. Have you heard of the Sehat Sahulat programme?

Yes

No (skip to Q42)

1. Are you eligible to enrol for it?

Yes

No (skip to Q42)

1. Have you enrolled to receive a Sehat health card?

Yes

No (skip to Q42)

1. On a scale from 1-10 with 10 being most difficult and 1 being easy, please select how you found enrolling onto the Sehat Sahulat programme.

Enter number:

1. Have you received your health card?

Yes

No (skip to Q42)

1. Have you used the card towards eye care treatment?

Yes

No (skip to question 40)

1. Would you have still accessed eye care and sought eye related treatment if the Sehat health card was not available to you?

Yes

No

1. Have you used the card to cover the cost of transport to reach a medical facility?

Yes

Used for only part of the cost

No

1. Since receiving the card on a scale from 1 – 10 how much more likely are you to go get your eyes checked with 1 being not likely and 10 being very likely.

Enter number:

**Overall experience with eye care service**

1. Which sector has better quality equipment and facilities:

Government

Private

1. How satisfied were you with the eye care professional that checked your eyes at your last visit?

Not satisfied

Neither dissatisfied nor satisfied

Satisfied

Very satisfied

1. Was the eye care professional that checked your eyes trained in:

Pakistan

United Kingdom

United Sates of America

Other

Unsure

1. How long did you have to wait in the eye care facility before getting seen at your last appointment?

Less than 15minutes

15-30 minutes

30 minutes-1 hour

1-2 hours

2-3 hours

3-4 hours

More than 4 hours

1. Did you book your last eye appointment in advance, or did you have to wait in a queue system on the day at you?

Booked in advance

Queue system for same day appointment

1. Overall, how satisfied were you with your overall experience with this eye care service?

Not satisfied

Neither dissatisfied or satisfied

v

Somewhat satisfied

v

Very satisfied

v

1. Out of the following options, please rank them from 1 to 5 based on their importance in improving current eye care services, with 1 being the most important and 5 being the least important.

_____ Increasing the availability of eye care services in rural areas

_____ Free eye care treatment

_____ Free transport to eye care services

_____ Shorter wait times in eye care facilities

_____ More lifts and ramps in eye care facilities

1. Please use the box below to type any additional comments about your experience with your local eye care services.

END OF SURVEY

**Part 2 (only for those who answered yes on question 8)**

1. Please select a reason(s) for not getting your eyes checked from the following list.

Not having any eye related issues

v

Cannot afford it

vvv

Unable to travel to an eye care facility

v

Don’t have time

v

Don’t know where to go to get my eyes checked

v

My eyes are not a health priority right now

v

Other, please specify

v

1. How often do you believe you should you get your eyes checked?

Every 1-3 months

v

Every 3-6 months

v

Every 6-12 months

v

Every 1 year-2 years

v

Every 2-3 years

v

More than 3 years

v

1. From the following list, what reason(s) prompted you to get an eye test for yourself today? (Can select more than one response)

Routine eye test

v

Change in level of vision

v

Needed an emergency eye check

v

To purchase or repair/replace spectacles

v

Contact lens check

v

Headaches

v

Family history of eye disease

v

Glaucoma monitoring

v

Cataract monitoring

v

Diabetic retinopathy monitoring

v

Monitoring of age-related macular degeneration

v

Advised by other healthcare provider

v

Other, please specify

v

1. How long did it take you to reach the hospital today?

Less than 15minutes

v

15-30 minutes

v

30 minutes – 1hour

vv

1-2 hours

v

2-3 hours

v

More than 3 hours

v

1. What mode of transportation do you use to get to the eye hospital today? (Can select more than one answer)

Motorcycle

v

Car

v

Rickshaw

v

Bus

v

Walk

v

1. Do you have any mobility issues?

Yes

v

No

v

1. Do you rely on someone to take you to this eye care service?

Yes

v

No

v

1. What is the most convenient day for you to get your eyes checked?

Monday – Friday

v

Weekends

v

Any day

v

1. What is the most convenient time for you to get your eyes checked?

Morning

v

Afternoon

v

Evening

v

Any time

v

**Cost of eye care**

1. Who pays for your eye appointment/treatments? (Can select more than one option)

Myself

v

Family

v

Health insurance

v

Other, please specify

v

1. Who pays for your travel costs to reach an eye care facility? (Can select more than one option)

Myself

v

Family

v

Health insurance

v

Other, please specify

v

1. **If you selected myself for any of the last two questions also answer this question, otherwise skip to Q13.**

Where did you use money from to cover these costs:

Income

v

Savings

v

Borrowed

v

Other, please specify

v

1. How much did you pay for your eye check-up today (appointment fee).

Rs.

1. How much did it cost you to travel to the eye hospital today?

Rs.

1. Have you heard of the Sehat Sahulat programme?

Yes

No (skip to Q24)

1. Are you eligible to enrol for it?

Yes

No (skip to Q24)

1. Have you enrolled to receive a Sehat health card?

Yes

No (skip to Q24)

1. On a scale from 1-10 with 10 being most difficult and 1 being easy, please select how you found enrolling onto the Sehat Sahulat programme.

Enter number:

1. Have you received your health card?

Yes

No (skip to Q24)

1. Have you used the card towards eye care treatment?

Yes

No (skip to Q22)

1. Would you have still accessed eye care and sought eye related treatment if the Sehat health card was not available to you?

Yes

No

1. Have you used the card to cover the cost of transport to reach a medical facility?

Yes

Used for only part of the cost

No

1. Since receiving the card on a scale from 1 – 10 how much more likely are you to go get your eyes checked with 1 being not likely and 10 being very likely.

Enter number:

1. Which sector has better quality equipment and facilities:

Government

v

Private

v

1. Out of the following options, please rank them from 1 to 5 based on their importance in improving current eye care services, with 1 being the most important and 5 being the least important.

_____ Increasing the availability of eye care services in rural areas

_____ Free eye care treatment

_____ Free transport to eye care services

_____ Shorter wait times in eye care facilities

_____ More lifts and ramps in eye care facilities

1. Please use the box below to type any additional comments about your experience with your local eye care services.

**END OF SURVEY**
