# Supplementary material for: Barriers to accessing eye care in Pakistan: a mixed methods study
Source: Prim Health Care Res Dev. 2025 Jul 15;26:e58. doi: 10.1017/S1463423625100261 (PMC12260732; doi:10.1017/S1463423625100261)
Supplement: Malik et al. supplementary material 3 — Malik et al. supplementary material [file S1463423625100261sup003.docx]

Supplementary file 3

Assumptions tested with statistical analysis and outcomes

1. There is a relationship between gender and awareness level of certain eye conditions.
2. There is a relationship between level of education and awareness level of certain eye conditions (i.e. health literacy).
3. There is a relationship between age and awareness level of certain eye conditions.
4. There is a relationship between community setting and awareness level of certain eye conditions.
5. There is a relationship between whether a previous eye test has been undertaken and awareness level of eye conditions.
6. There is a relationship between gender and eye test interval.
7. There is a relationship between level of education and eye test interval.
8. There is a relationship between age and eye test interval.
9. There is a relationship between community setting and eye test interval.
10. There is a relationship between employment status and eye test interval.
11. There is a relationship between gender and the sector visited for an eye test.
12. There is a relationship between level of education and the sector visited for an eye test.
13. There is a relationship between age and the sector visited for an eye test.
14. There is a relationship between community setting and the sector visited for an eye test.
15. There is a relationship between employment status and the sector visited for an eye test.
16. There is a relationship between gender and requiring support to reach an eye care facility.
17. There is a relationship between level of education and requiring support to reach an eye care facility.
18. There is a relationship between age and requiring support to reach an eye care facility.
19. There is a relationship between community setting and requiring support to reach an eye care facility.
20. There is a relationship between employment status and requiring support to reach an eye care facility.
21. There is a relationship between where an eye care professional was trained and the cost of an eye test.
22. There is a relationship between community setting and the cost of an eye test.
23. There is a relationship between gender and awareness of the SSP.
24. There is a relationship between level of education and awareness of the SSP.
25. There is a relationship between age and awareness of the SSP.
26. There is a relationship between community setting and awareness of the SSP.
27. There is a relationship between employment status and awareness of the SSP.
28. There is a relationship between gender and eligibility to enrol to receive the Sehat Insaf Card.
29. There is a relationship between level of education and eligibility to enrol to receive the Sehat Insaf Card.
30. There is a relationship between age and eligibility to enrol to receive the Sehat Insaf Card.
31. There is a relationship between community setting and eligibility to enrol to receive the Sehat Insaf Card.
32. There is a relationship between employment status and eligibility to enrol to receive the Sehat Insaf Card.
33. There is a relationship between eye care sector and where an eye care professional was trained.
34. There is a relationship between participants wait time and overall level of satisfaction with eye care service.
35. There is a relationship between wait times and whether a queue system was used or booking in advance.
36. There is a relationship between wait times and type of service utilised (government or private).

P-values from chi square test, Kruskal Wallis and Mann Whitney U

| **Dependent variables** | **Independent variables** | | | | | | | | | |
| --- | --- | --- | --- | --- | --- | --- | --- | --- | --- | --- |
|  | Age | Gender | Education level | Community setting | Employment status | Previous eye exam | Sector | Location of training of eye care professional | Satisfaction level | Queue system vs. booking in advance |
| Awareness of cataract | Chi^2^ p=0.492 | Chi^2^ p=1.597 | Chi^2^ **p=0.000*** | Chi^2^ p=0.123 |  |  |  |  |  |  |
| Awareness of refractive error | Chi^2^ **p=0.014*** | Chi^2^ p=0.289 | Chi^2^ p=0.906 | Chi^2^ p=0.377 |  |  |  |  |  |  |
| Awareness of glaucoma | Chi^2^ p=0.462 | Chi^2^ p=0.914 | Chi^2^ p=0.055 | Chi^2^ p=0.055 |  |  |  |  |  |  |
| Awareness of eye problems caused by diabetes | Chi^2^ p=0.085 | Chi^2^ p=0.259 | Chi^2^ p=0.147 | Chi^2^ p=0.527 |  |  |  |  |  |  |
| Awareness of dry eye | Chi^2^ p=0.269 | Chi^2^ p=0.239 | Chi^2^ p=0.121 | Chi^2^ **p=0.012*** |  |  |  |  |  |  |
| Awareness of macular degeneration | Chi^2^ p=0.790 | Chi^2^ p=0.202 | Chi^2^ p=0.695 | Chi^2^ p=0.812 |  |  |  |  |  |  |
| Total number of eye conditions aware of | Mann-Whitney U  p=0.411 | Kruskal-Wallis  p=0.322 | Mann-Whitney U  p=0.613 | Kruskal-Wallis  p=0.393 |  | Kruskal-Wallis  p=0.133 |  |  |  |  |
| Previous eye exam | Chi^2^ p=0.067 | Chi^2^ p=0.088 | Chi^2^ **p=0.002*** | Chi^2^ p=0.087 |  |  |  |  |  |  |
| How often one believes they should get eyes checked | Chi^2^ **p=0.047*** | Chi^2^ p=0.259 | Chi^2^ p=0.185 | Chi^2^ p=0.507 | Chi^2^  p=0.563 |  |  |  |  |  |
| Sector visited | Chi^2^ p=0.717 | Chi^2^ p=0.865 | Chi^2^ **p=0.001*** | Chi^2^ p=0.093 | Chi^2^ **p=0.029*** |  |  |  |  |  |
| Mobility issues | Chi^2^ p=0.752 | Chi^2^ **p=0.027*** | Chi^2^ p=0.238 | Chi^2^ p=0.656 | Chi^2^ p=0.888 |  |  |  |  |  |
| Support to reach eye care service | Chi^2^ p=0.134 | Chi^2^ **p=0.003*** | Chi^2^ **p=0.042*** | Chi^2^ p=0.570 | Chi^2^ **p=0.001*** |  |  |  |  |  |
| Cost of eye test |  |  |  | Mann-Whitney U  **p=0.006*** |  |  |  | Mann-Whitney U  **p=0.010*** |  |  |
| Awareness of SSP | Chi^2^ p=0.970 | Chi^2^ p=0.081 | Chi^2^ p=0.096 | Chi^2^ p=0.656 | Chi^2^ p=0.922 |  |  |  |  |  |
| Eligibility to enrol into SSP | Chi^2^ p=0.314 | Chi^2^ **p=0.009*** | Chi^2^ p=0.431 | Chi^2^ p=0.276 | Chi^2^ p=0.104 |  |  |  |  |  |
| Location of training of eye care professional |  |  |  |  |  |  | Chi^2^ p=0.116 |  |  |  |
| Wait time |  |  |  |  |  |  | Chi^2^ p=0.539 |  | Chi^2^ p=0.102 | Chi^2^ p=0.372 |

**Table 1:** Results of binary logistic regression for level of education and awareness of cataract

| Education Level | | $\boldsymbol{\beta}$ | S.E. | P | Exp($\boldsymbol{\beta}$)  (Odds ratio) | 95% C.I.for Exp($\boldsymbol{\beta}$) (odds ratio) | |
| --- | --- | --- | --- | --- | --- | --- | --- |
|  |  |  |  |  |  | Lower | Upper |
|  | Less than high school (vs.) |  |  |  |  |  |  |
|  | High school | -1.263 | .682 | .064 | .283 | .074 | 1.076 |
|  | More than high school | -1.811 | .459 | **.000*** | .163 | .066 | .402 |

β ‐regression co‐efficient; S.E.‐standard error; P- *P-*value; C.I.‐confidence interval; Exp(β)- exponential beta

**Table 2:** Results of binary logistic regression comparing age and level of education against whether a previous eye test had been undertaken or not.

|  |
| --- |

| Independent variables | | $\boldsymbol{\beta}$ | S.E. | P | OR | 95% C.I.for OR | |
| --- | --- | --- | --- | --- | --- | --- | --- |
|  |  |  |  |  |  | Lower | Upper |
|  | Age |  |  |  |  |  |  |
|  | Less than 50 years (vs.) |  |  |  |  |  |  |
|  | 51-60 years | 1.436 | .527 | **.006*** | 4.205 | 1.496 | 11.817 |
|  | More than 61 years | .891 | .529 | .092 | 2.437 | .863 | 6.879 |
|  | Education level |  |  |  |  |  |  |
|  | Less than high school (vs.) |  |  |  |  |  |  |
|  | High school | 1.368 | .775 | .077 | 3.929 | .860 | 17.945 |
|  | More than high school | 1.725 | .483 | **.000*** | 5.615 | 2.179 | 14.465 |

**Table 3:** Multinomial logistic regression for age and perceived interval of eye test (1 to 6 months). Comparison category is 1 to 2 years.

| Interval | Age | $\boldsymbol{\beta}$ | S.E. | P | OR | 95% C.I.for OR | |
| --- | --- | --- | --- | --- | --- | --- | --- |
|  |  |  |  |  |  | Lower | Upper |
| 1-6 months | More than 61 years (vs.) |  |  |  |  |  |  |
|  | 51-60 years | .674 | .587 | .251 | 1.962 | .621 | 6.193 |
|  | Less than 50 years | 1.974 | .671 | **.003*** | 7.200 | 1.933 | 26.812 |

**Table 4:** Results from multinomial logistic regression for level of education, employment status and type of service used. The comparison category is private service.

| Dependent variables (Type of service) | Independent variables | | 𝜷 | S.E. | P | OR | 95% C.I.for OR | |
| --- | --- | --- | --- | --- | --- | --- | --- | --- |
|  |  |  |  |  |  |  | Lower | Upper |
| Government |  | Employment status |  |  |  |  |  |  |
|  |  | Employed (vs.) |  |  |  |  |  |  |
|  |  | Unemployed | -0.055 | 0.506 | 0.914 | 0.947 | 0.352 | 2.550 |
|  |  | Education level |  |  |  |  |  |  |
|  |  | More than high school (vs.) |  |  |  |  |  |  |
|  |  | High school | 1.887 | .601 | **.002*** | 6.600 | 2.033 | 21.431 |
|  |  | Less than high school | -.981 | 1.093 | .370 | .375 | .044 | 3.196 |
| Charity |  | Employment status |  |  |  |  |  |  |
|  |  | Employed (vs.) |  |  |  |  |  |  |
|  |  | Unemployed | 1.718 | 0.853 | **0.044*** | 5.576 | 1.047 | 29.690 |
|  |  | Education level |  |  |  |  |  |  |
|  |  | More than high school (vs.) |  |  |  |  |  |  |
|  |  | High school | 2.434 | .786 | **.002*** | 11.400 | 2.443 | 53.188 |
|  |  | Less than high school | 1.047 | .918 | .254 | 2.850 | .472 | 17.225 |

**Table 5:** Results from binary logistic regression for gender, employment status, level of education, and whether the respondent relied on someone else to reach an eye care facility.

| Independent variables | | 𝜷 | S.E. | P | OR | 95% C.I.for OR | |
| --- | --- | --- | --- | --- | --- | --- | --- |
|  |  |  |  |  |  | Lower | Upper |
|  | Gender |  |  |  |  |  |  |
|  | Males (vs.) |  |  |  |  |  |  |
|  | Females | .933 | .452 | .039 | 2.541 | 1.048 | 6.162 |
|  | Employment status |  |  |  |  |  |  |
|  | Unemployed (vs.) |  |  |  |  |  |  |
|  | Employed | -.655 | .477 | .170 | .520 | .204 | 1.322 |
|  | Education level |  |  |  |  |  |  |
|  | More than high school (vs.) |  |  |  |  |  |  |
|  | High school | -.642 | .800 | .422 | .526 | .110 | 2.522 |
|  | Less than high school | -.929 | .550 | .091 | .395 | .134 | 1.160 |

**Table 6:** Results of binary logistic regression for gender and eligibility to enrol for the Sehat Insaf Card

| Gender | | $\boldsymbol{\beta}$ | S.E. | P | OR | 95% C.I.for OR | |
| --- | --- | --- | --- | --- | --- | --- | --- |
|  |  |  |  |  |  | Lower | Upper |
|  | Male (vs.) |  |  |  |  |  |  |
|  | Female | 1.126 | .436 | **.010*** | 3.083 | 1.311 | 7.251 |
