# Supplementary material for: Barriers to accessing eye care in Pakistan: a mixed methods study
Source: Prim Health Care Res Dev. 2025 Jul 15;26:e58. doi: 10.1017/S1463423625100261 (PMC12260732; doi:10.1017/S1463423625100261)
Supplement: Malik et al. supplementary material 4 — Malik et al. supplementary material [file S1463423625100261sup004.docx]

Supplementary file 4

Survey responses for continuous variables and tests of normality

| **Survey question** | **Number of participant responses** | **Mean ± SD** | **Median, range** | **Shapiro-Wilk** | |
| --- | --- | --- | --- | --- | --- |
|  |  |  |  | **Statistic** | **Sig.** |
| Estimate the amount you paid for your last check-up | 92 | 1563.92 ± 2082.23 PKR | 1000PKR, 0-15000PKR | 0.636 | 0.000 |
| How much did you pay for your eye check today? (Al-Shifa Eye Trust) | 30 | 305PKR ± 382 PKR | 400PKR, 0-15000PKR | 0.670 | 0.000 |
| How much did you pay for your eye check today? (THQ Chakwal) | 15 | 23.33±90.37 PKR | 0, 0-350 PKR | 0.284 | 0.000 |
| Estimate the amount you spent on eye care treatment (online only). | 72 | 14668.75 ± 40181.18 PKR | 4750 PKR, 0-300000PKR | 0.344 | 0.000 |
| Estimate the total amount in PKR that you have spent on travel costs to reach an eye care facility. | 100 | 1221.60 ±2400.30 PKR | 375PKR, 0-12000PKR | 0.493 | 0.000 |
| How much did it cost you to travel to the eye hospital today? (Al-Shifa) | 30 | 768.33 ± 1049.92 PKR | 300PKR, 0-5000PKR | 0.933 | 0.300* |
| How much did it cost you to travel to the eye hospital today? (THQ Chakwal) | 15 | 240.67 ± 138.22 PKR | 200PKR, 0-500PKR | 0.627 | 0.000 |
| Did you find the eye care service you used affordable? Rate its affordability on a scale from 1-10 with 1 being not affordable and 10 being very affordable. | 108 | 5.77 ± 3.35 | 6, 1-10 |  |  |
| On a scale from 1-10 with 10 being most difficult and 1 being easy, please select how you found enrolling onto the Sehat Sahulat programme. | 14 | 4.36 ± 2.90 | 5, 1-10 |  |  |
| Since receiving the card on a scale from 1 – 10 how much more likely are you to go get your eyes checked with 1 being not likely and 10 being very likely. | 5 | 4.40 ± 3.72 | 5, 1-10 |  |  |
| Out of the following options, please rank them from 1 to 5 based on their importance in improving current eye care services, with 1 being the most important and 5 being the least important: |  |  |  |  |  |
| - Increasing the availability of eye care services in rural areas | 31 | 1.71 ± 0.82 | 1, 1-3 |  |  |
| - More lifts and ramps in eye care facilities | 31 | 2.10 ± 0.91 | 2, 1-4 |  |  |
| - Free eye care treatment | 31 | 2.39 ±0.88 | 2, 1-5 |  |  |
| - Shorter wait times in eye care facilities | 31 | 4.32 ± 0.79 | 4, 1-5 |  |  |
| - Free transport to eye care services | 31 | 4.45 ± 0.72 | 5, 2-5 |  |  |

Survey responses for categorical variables (proportion of all participants, absolute number in brackets)

| Ability to perceive | | | | | | | | | | | | | | | |  |
| --- | --- | --- | --- | --- | --- | --- | --- | --- | --- | --- | --- | --- | --- | --- | --- | --- |
| Question Topic | **Responses** | | | | | | | | | | | | | | |  |
| Awareness of eye condition | Refractive error | Cataract | Eye issues secondary to diabetes | Glaucoma | Macular degeneration | Dry eye |  |  |  |  |  | |  | |  |  |
|  | 59.5%  (97/163) | 54.0%  (88/163) | 35.0%  (57/163) | 31.9%  (52/163) | 14.7%  (24/163) | 14.7%  (24/163) |  |  |  |  |  | |  | |  |  |
| Previous eye test | Yes | No |  |  |  |  |  |  |  |  |  | |  | |  |  |
|  | 75.5%  (123/163) | 24.5%  (40/163) |  |  |  |  |  |  |  |  |  | |  | |  |  |
| Reasons for having eyes tested | Change in level of vision | Diabetic retinopathy monitoring | Routine eye test | To purchase or repair spectacles | Headaches | Family history of eye disease | Emergency eye check | Cataract monitoring | ARMD monitoring | Contact lens check | Advised by other healthcare practitioner | | Glaucoma monitoring | | Other |  |
|  | 40.8%  (58/142) | 26.8%  (38/142) | 18.3%  (26/142) | 14.1%  (20/142) | 11.3%  (16/142) | 7.7%  (11/142) | 6.3%  (9/142) | 4.9%  (7/142) | 4.2%  (6/142) | 2.8%  (4/142) | 2.8%  (4/142) | | 0.7%  (1/142) | | 0.7%  (1/142) |  |
| Reasons for not having eyes tested | No eye related issues | Unable to take time out to get eyes checked | Unsure where to go to get eyes checked | Unable to afford care | Unable to travel to eye care facility | Eyes not a health priority |  |  |  |  |  | |  | |  |  |
|  | 65.0%  (26/40) | 20.0%  (8/40) | 7.5%  (3/40) | 5.0%  (2/40) | 5.0%  (2/40) | 5.0%  (2/40) |  |  |  |  |  | |  | |  |  |
| Reasons for not having eye test within 2 years | No change in vision | Don’t have time | Don’t attend regular checks | Not made next appointment | Eyes not health priority | Cannot afford it | Cannot travel | Other |  |  |  | |  | |  |  |
|  | 61.3%  (19/31) | 61.3%  (19/31) | 58.1%  (18/31) | 32.3%  (10/31) | 16.1%  (5/31) | 3.2%  (1/31) | 3.2%  (1/31) | 9.7%  (3/31) |  |  |  | |  | |  |  |
| Time since last eye test | Less than 3 months | 3-6 months | 6-12 months | 1-2 years | 2-3 years | More than  3 years |  |  |  |  |  | |  | |  |  |
|  | 20.5%  (25/122) | 18.9%  (23/122) | 18.0%  (22/122) | 17.2%  (21/122) | 7.4%  (9/122) | 18.0%  (22/122) |  |  |  |  |  | |  | |  |  |
|  | 1-3 months | 3-6 months | 6-12 months | 1-2 years | 2-3 years | More than 3 years |  |  |  |  |  | |  | |  |  |
| Interval between one eye check to the next | 12.3%  (15/122) | 12.3%  (15/122) | 30.3%  (37/122) | 23.8%  (29/122) | 9.0%  (11/122) | 12.3%  (15/122) |  |  |  |  |  | |  | |  |  |
| How often do you believe you should get your eyes checked? | 14.1%  (20/142) | 21.1%  (30/142) | 38.0%  (54/142) | 22.5%  (32/142) | 2.8%  (4/142) | 1.4%  (2/142) |  |  |  |  |  | |  | |  |  |
| Ability to reach | | | | | | | | | | | | | | | |  |
| Previously visited service for eye test | Private | Government | Charity | Military | Other |  |  |  |  |  |  |  | |  | | |
|  | 57.9%  (70/121) | 25.6%  (31/121) | 9.9%  (12/121) | 4.1%  (5/121) | 2.5%  (3/121) |  |  |  |  |  |  |  | |  | | |
|  | Less than 15 minutes | 15-30 minutes | 30-60 minutes | 1-2 hours | 2-3 hours | More than  3 hours |  |  |  |  |  |  | |  | | |
| Time to reach previous eye care service | 14.0%  (17/121) | 34.7%  (42/121) | 29.8%  (36/121) | 13.2%  (16/121) | 4.1%  (5/121) | 5.0%  (6/121) |  |  |  |  |  |  | |  | | |
| Time taken to reach eye hospital today (Al-Shifa Trust) |  | 23.3%  (7/30) | 20.0%  (6/30) | 30.0%  (9/30) | 20.0%  (6/30) | 6.7%  (2/30) |  |  |  |  |  |  | |  | | |
| Time taken to reach eye hospital today (Kallar Kahar THQ) | 6.7%  (1/50) | 33.3%  (5/15) | 33.3%  (5/15) | 26.7%  (4/15) |  |  |  |  |  |  |  |  | |  | | |
|  | Motorcycle | Car | Rickshaw | Bus | Walk |  |  |  |  |  |  |  | |  | | |
| Mode of transport to reach (previously) visited eye care facility | 11.4%  (14/123) | 62.6%  (77/123) | 8.9%  (11/123) | 13.8%  (17/123) | 4.9%  (6/123) |  |  |  |  |  |  |  | |  | | |
| Mode of transport to reach eye hospital today (Al-Shifa & Kallar Kahar only) | 8.9%  (4/45) | 37.8%  (17/45) | 13.3%  (6/45) | 37.8%  (17/45) | 2.2%  (1/45) |  |  |  |  |  |  |  | |  | | |
|  | Yes | No |  |  |  |  |  |  |  |  |  |  | |  | | |
| Mobility issues present | 14.2%  (20/141) | 85.8%  (121/141) |  |  |  |  |  |  |  |  |  |  | |  | | |
| Required support to reach eye care service | 32.5%  (40/123) | 67.5%  (83/123) |  |  |  |  |  |  |  |  |  |  | |  | | |
| Required support to reach eye care service (Al-Shifa Trust) | 70.0%  (21/30) | 30.0%  (9/30) |  |  |  |  |  |  |  |  |  |  | |  | | |
| Required support to reach eye care service (Kallar Kahar THQ) | 13.3%  (2/15) | 86.7%  (13/15) |  |  |  |  |  |  |  |  |  |  | |  | | |
| Preferred day for eye test | Any day | Weekday | Weekend |  |  |  |  |  |  |  |  |  | |  | | |
|  | 53.9%  (76/141) | 17.0%  (24/141) | 29.1%  (41/141) |  |  |  |  |  |  |  |  |  | |  | | |
| Preferred time for eye test | Morning | Afternoon | Evening | Anytime |  |  |  |  |  |  |  |  | |  | | |
|  | 47.2%  (67/142) | 12.7%  (18/142) | 22.5%  (32/142) | 17.6%  (25/142) |  |  |  |  |  |  |  |  | |  | | |
| Ability to pay | | | | | | | | | | | | | | | |  |
|  | Myself | Family | Health insurance | Other |  |  |  |  |  |  |  |  | |  | | |
| Who pays for your eye appointment / treatment? | 54.2%  (78/144) | 36.8%  (53/144) | 4.2%  (6/144) | 4.9%  (7/144) |  |  |  |  |  |  |  |  | |  | | |
| Who pays for travel costs? | 59.7%  (83/139) | 36.7%  (51/139) | 1.4%  (2/139) | 2.2%  (3/139) |  |  |  |  |  |  |  |  | |  | | |
| Where did you use money from to cover these costs? | Income | Savings | Borrowed | Other |  |  |  |  |  |  |  |  | |  | | |
|  | 85.5%  (71/83) | 15.7%  (13/83) | 2.4%  (2/83) | 2.4%  (2/83) |  |  |  |  |  |  |  |  | |  | | |
|  | Yes | No |  |  |  |  |  |  |  |  |  |  | |  | | |
| Have you heard of the SSP? | 63.5% (101/159) | 35.5% (58/159) |  |  |  |  |  |  |  |  |  |  | |  | | |
| Eligibility for SSP enrolment | 49.5% (49/99) | 50.5% (50/99) |  |  |  |  |  |  |  |  |  |  | |  | | |
| Have you enrolled to receive a health card? | 38.3% (18/47) | 61.7% (29/47) |  |  |  |  |  |  |  |  |  |  | |  | | |
| Received SSP card | 41.2% (7/17) | 58.8% (10/17) |  |  |  |  |  |  |  |  |  |  | |  | | |
| Used SSP card towards eye care treatment | 14.3% (1/7) | 85.7% (6/7) |  |  |  |  |  |  |  |  |  |  | |  | | |
| Would you still have accessed eye care and sought eye related treatment if the SSP card was not available to you? |  | 100% (1/1) |  |  |  |  |  |  |  |  |  |  | |  | | |
| Used SSP card to cover the cost of transport to reach medical facility |  | 100% (7/7) |  |  |  |  |  |  |  |  |  |  | |  | | |
| Suitability of eye care service | | | | | | | | | | | | | | | |  |
| Which sector has better quality equipment and facilities? | Government | Private |  |  |  |  |  |  |  |  |  |  | |  | | |
|  | 41.0% (57/139) | 59.0% (82/139) |  |  |  |  |  |  |  |  |  |  | |  | | |
| Wait time in eye care facility | Less than 15 minutes | 15-30 minutes | 30-60 minutes | 1-2 hours | 2-3 hours | More than 4 hours |  |  |  |  |  |  | |  | | |
|  | 0.8%  (1/120) | 44.2% (53/120) | 37.5% (45/120) | 13.3% (16/120) | 3.3%  (4/120) | 0.8% (1/120) |  |  |  |  |  |  | |  | | |
| Booked appointment or waited in queue system on the day | Booked | Queue system |  |  |  |  |  |  |  |  |  |  | |  | | |
|  | 54.5% (66/121) | 45.5% (55/121) |  |  |  |  |  |  |  |  |  |  | |  | | |
| Country eye care professional was trained in | Pakistan | UK | USA | Unsure | Other |  |  |  |  |  |  |  | |  | | |
|  | 45.1% (55/122) | 12.3% (15/122) | 6.6% (8/122) | 33.6% (41/122) | 2.5% (3/122) |  |  |  |  |  |  |  | |  | | |
|  | Not satisfied | Neither satisfied or dissatisfied | Somewhat satisfied | Very satisfied |  |  |  |  |  |  |  |  | |  | | |
| Level of satisfaction with the eye care professional that checked eyes at last visit | 4.1%  (5/121) | 9.9% (12/121) | 47.9% (58/121) | 38.0% (46/121) |  |  |  |  |  |  |  |  | |  | | |
| Level of satisfaction with overall experience with (previous) eye care service | 0.8 (1/119) | 9.2 % (11/119) | 47.0% (56/119) | 42.9% (51/119) |  |  |  |  |  |  |  |  | |  | | |
